# Gingival Recession after Surgical Endodontic Treatment and Quality of Life: A Systematic Review and Meta-analysis

**DOI:** 10.3290/j.ohpd.b1176847

**Published:** 2021-04-09

**Authors:** Ruaa A. Alamoudi, Nuha S. Alghamdi, Saad M. Alqahtani, Rana A. S. Alamoudi, Khlood Baghlaf

**Affiliations:** a Assistant Professor, Endodontic Department, Faculty of Dentistry, King Abdulaziz University, Jeddah, Saudi Arabia. Study concept and design, data analysis, critically revised the manuscript.; b Associate Professor, Endodontic Department, Faculty of Dentistry, King Khalid University, Abha 62529, Saudi Arabia. Study design, data acquisition, wrote the manuscript.; c Associate Professor and Chair, Periodontic Department, Faculty of Dentistry, King Khalid University, Abha, Saudi Arabia. Study concept, data acquisition and analysis, critically revised the manuscript.; d Assistant Professor, Pediatric Dentistry Department, Faculty of Dentistry, King Abdulaziz University, Jeddah, Saudi Arabia. Study concept, data acquisition and analysis, wrote the manuscript.; e Assistant Professor, Pediatric Dentistry Department, Faculty of Dentistry, King Abdulaziz University, Jeddah, Saudi Arabia. Study concept and designm data acquisition, critically revised the manuscript.

**Keywords:** flap incision, gingival recession, oral health quality of life, surgical endodontic treatment, systematic review

## Abstract

**Purpose:**

This systematic review addressed flap designs in endodontic surgery which can have an impact on the Oral Health Related Quality of Life (OHRQoL).

**Materials and Methods:**

Four electronic databases were searched (PubMed, Cochrane Library, Web of Science, and Scopus) to identify all studies up to November 2019 that investigated the effect of flap designs on gingival recession and quality of life among healthy adults.

**Results:**

The initial search identified 2701 references. Ten studies were included in this systematic review; two were randomised clinical trials and eight were non-randomised clinical trials. Studies showed that sulcular incision increases the risk of gingival recession and decreases OHRQoL. Two studies were included in the meta-analysis in relation to gingival recession. The pooled results demonstrated that submarginal incision showed a decreased weighted mean difference in gingival recession by 0.31 mm (95% CI: 0.12 – 0.51) (p = 0.002) compared to sulcular incision.

**Conclusion:**

Sulcular incision flap unfavourably affect the level of gingiva and OHRQoL. All nonrandomised studies had a statistically significant bias and the sample sizes in all studies were relatively small. More gingival recession and lower OHRQoL were associated with sulcular incision. Additional investigations are warranted to provide more evidence.

Surgical endodontics has been recently introduced and the paradigm has shifted from mere elimination of periapical pathology to the successful accomplishment of aspects concerning function, aesthetics and preservation of surrounding periodontal structures.^26^

The flap incision requires reflection of the gingival tissue to expose the bone covering the root(s) and the apices in order to treat the apical pathology. A variety of flap incisions have been tried and utilised.^3,51,57^ Intra-sulcular incisions appear to be the preferable design as they enhance site visibility, and allow easy suturing and tissue handling. Recently, submarginal incision has been introduced to overcome certain limitations associated with intra-sulcular incision, such as gingival recession, longer surgical duration, excessive tissue manipulation, and difficulty in flap closure. However, this design hinders the visibility of the surgical site.^36,57^ Thereafter, the papilla preservation flap was proposed to accomplish better visualisation with healthier primary wound closure, preventing gingival recession and tissue necrosis.^9,10^

Various complications related to the different incision techniques have been reported in the literature. They can be summarised mainly as gingival recession,^20,44^ post-operative pain, inflammation, hampered mastication and impaired speech. Gingival recession increases the risk of erosion, abrasion, attrition and abfraction, altering the functional and aesthetic concerns, dentinal hypersensitivity, and root caries.^7^ A proper selection of flap design will help minimise post-operative complications and result in a favourable outcome.

Post-operative pain and swelling have a significant effect on the quality of life (QoL). It reflects the goodness of life, as subjectively evaluated by the quality of life experience and objectively judged by assessment of external circumstances.^39^ The World Health Organization (WHO) defines QoL as ‘an individual’s perception of their position in life in the context of the culture and value systems in which they live and in relation to their goals, expectations, standards and concerns.’^8^ Patients’ QoL plays a significant role by helping us evaluate the significance of an illness or disease affecting their daily life. It is not only associated with the severity of the disease, but also a patient’s experience with the contingencies of the disease and treatment consequences. In dental practice, QoL related to oral health has been recently employed as an essential aspect to assess dental treatment outcomes.^23,33^ Inglehart and Bagramian^24^ defined OHRQoL as ‘the absence of negative impacts of oral conditions on social life and a positive sense of dento-facial self-confidence.’

Since endodontic surgery adversely influences gingival recession and OHRQoL of the patients, the aims of this systematic review were to answer the following questions: 1) Do different flap designs in endodontic surgery have an impact on gingival recession and gingival aesthetics? 2) Do different flap designs in endodontic surgery have an impact on the OHRQoL?

This review will offer more in-depth knowledge regarding the impact of different flap designs on gingival recession and OHRQoL of patients who underwent periapical endodontic surgery.

## Materials and Methods

The protocol was registered on PROSPERO (www.crd.york.ac.uk/prospero; CRD42019155488). The inclusion criteria were: studies assessing the impact of different flap designs on the gingival recession, quality of life and/or patient satisfaction among healthy adult patients undergoing endodontic surgery. Studies were excluded if they were done on pediatric patients or investigated non-surgical endodontics.

### Study Design

This systematic analysis included randomised clinical trials (RCTs), non-randomised trials, prospective cohort studies, case-control studies, and cross-sectional studies that assessed the gingival recession, quality of life and/or patient satisfaction after endodontic surgery with different flap designs. Editorial letters, pilot studies, historical reviews, literature review, in vitro studies and descriptive studies such as case reports and case series were excluded.

### Search and Data Extraction

The following databases were searched to identify all related articles up to November 2019 without language restrictions: PubMed, the Cochrane Central Register of Controlled Trials, Web of Science, and Scopus. Other databases, e.g. EMBASE, OVID, and Google-Scholar, were excluded because they showed the same results. The search strategy included the terms related to PICOS elements. The key words used for the search were ‘endodontic surgery’, ’periradicular/periapical surgery’, ‘apical surgery’, ’flap design’, ‘gingival recession’, ‘esthetics’, ‘quality of life’, and ‘patient satisfaction’.

The titles and abstracts of the studies reviewed using the search strategy as well as those from additional sources were screened independently by two reviewers considering the above-mentioned selection criteria. If the title and abstract provided insufficient information, the decision for inclusion was based on full-text screening. All the searched studies were imported to reference management software and checked for duplicates. The full text of eligible studies was retrieved and assessed by two reviewers independently. Any disagreement between the two reviewers was resolved through discussion involving a third reviewer.

Data extraction included: (1) study design; (2) sample size and demographics; (3) intervention: type of flap design used in endodontic surgery; (4) follow-up period; (5) confounding variables; (6) outcomes (primary and secondary).

### Quality Assessment

Two reviewers independently assessed the quality of the study methodologies included. For randomised clinical trials, the Cochrane Collaborations Risk Bias Tool was used. The following domains were assessed: a sequence generation, allocation concealment, building of outcome assessors, incomplete outcome data, selective reporting and other biases. Studies with low or unclear risk of bias were to be included in the meta-analysis. The authors of the included studies were contacted for clarification, if required. Nonrandomised clinical studies were assessed using the Newcastle Ottawa Scale (NOS), and studies with good methodology (more than five stars on the NOS) were eligible for meta-analysis.

Meta-analysis was performed using random models and all statistical analyses were undertaken using Review Manager v 5.1 (Nordic Cochrane Centre, Cochrane Collaboration, 2001). At least ten studies should be included in a meta-analysis to assess the publication bias. Statistical heterogeneity was assessed by inspecting a graphic display of the estimated exposure effects from individual trials, with associated 95% confidence intervals. Heterogeneity was quantified using I^2^, in which values above 50% indicate moderate to high heterogeneity, which might preclude meta-analysis. A weighted treatment effect was to be calculated, and the results for gingical recession were expressed as mean differences.

## Results

### Study Identification

**Fig 1:**
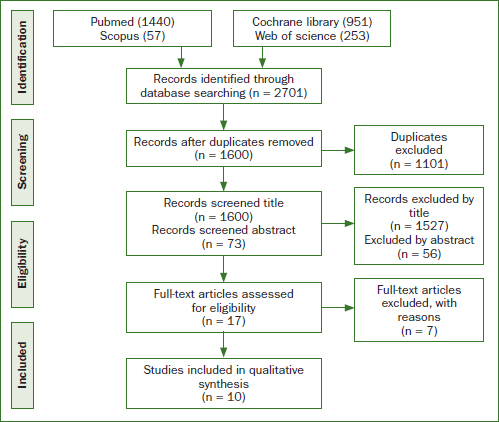
PRISMA flow diagram shows the number of articles identified at each stage of the study.

The initial search identified 2701 references in the electronic databases: 1440 from PubMed, 951 from the Cochrane library, 253 from Web of Science, and 57 from Scopus. The literature search was restricted to these search engines as the exploration of the others produced same articles. The manual search on the topic yielded no additional relevant articles. After removing duplicates, 1600 references were eligible for title screening. Seventy-three references were eligible for inclusion based on their abstracts, and 17 references were subject to full-text evaluation (Fig 1). Following the full-text evaluation, seven articles were excluded.^11,15,27,34,35,37,40^

The reasons for exclusion are summarised in Table 1. Eventually, ten articles^2,11,13,19,27,46,47,53,54,56^ – two randomised clinical trials (RCTs) and eight non-randomised clinical studies – were subjected to data extraction, quality assessment, data synthesis and analysis. Using the Kappa statistic, inter-observer agreement regarding article selection was κ = 0.85, indicating perfect agreement between the reviewers. Table 2 summarises the characteristics of the studies included. All studies were conducted in Europe, except one of Asian origin.^2^ All studies were clinical trials with sample sizes ranging from 12 to 81 patients, including both males and females. The lower age limit of patients in all studies was 18 years, except in two studies, with two under 18 years^28,37^ and another that did not mention the age.^56^ Five articles drew comparisons between papilla-based incisions and sulcular (marginal or intra-sulcular) incisions.^11,46,47,53,54^ Two articles compared the submarginal incision with the sulcular (marginal or intra-sulcular) incision.^2,27^ One study compared three types of incision: papilla-based, submarginal, and sulcular.^56^ One study evaluated the marginal incision without a control group^19^ and another study compared gingival flap with semilunar flap without mentioning the type of incision.^13^ Follow-up periods ranged from 1 h to 7 days in studies that assessed the quality of life,^2,12,13^ and from 0 to 12 months in studies that evaluated the gingival recession.^2,4,19,27,46,53,54,56^ Five studies discussed preoperative medication, such as analgesics, 0.2% chlorhexidine, antibiotics, or corticosteroid supplements.^2,27,47,53,54^ Seven studies reported postoperative, care including cold compresses, analgesics, 0.2% chlorhexidine, and antibiotics.^11,13,46,47,53,54,56^ Four studies reported smoking before surgery as a confounding factor.^11,46,47,56^

**Table 1:** Studies excluded from the analysis after full-text reading and exclusion criteria

Study	Reason for exclusion
Del Fabbro et al, 2012	Investigated the effect of platelet concentration in endodontic surgery
Esser et al, 1986	Not written in English
Kreisler et al, 2004	Investigated the effect of low-level laser laser in endodontic surgery
Meschi et al, 2018	Investigated the effect of platelet-rich fibrin in endodontic surgery
Metin et al, 2018	Investigated the effect of low-level laser laser in endodontic surgery
Payer et al, 2005	Investigated the effect of low-level laser laser in endodontic surgery
Rixecker et al, 1986	Not written in English

**Table 2:** Characteristics of included studies

Study	Type of study	Country	Sample Size (n)	Type of flaps	Outcomes/outcomemeasures	+/0/-	Follow-up duration	Confounder
Grung, 1973	NRCT	Denmark	n =15 F (n=7) M(n=8) 14-40Y	Marginal incision	Amount of recession Plaster model	GR more with marginal incision	3 Mo	
Velvart et al, 2003	NRCT	Switzerland	n =12 F (n=6) M(n=6) 36-63Y	1. Papilla-based incision 2. Sulcular incision	Amount of recession Plaster model	At 1 month PBI: 0.07 ± 0.09 mm SI: 1.10 ± 0.72 mm At 3 months PBI: 0.10 ± 0.15 mm SI: 1.25 ± 0.81 mm	1 Mo 3 Mo	Cold compress NSAID 0.2% CHX
Velvart et al, 2004	NRCT	Switzerland	n=12 F (n=6) M (n=6) 36-63Y	1. Papilla-based incision 2. Sulcular incision	Amount of recession Plaster model	At 12 months PBI: 0.06 ± 0.21 mm SI: 0.98 ± 0.75 mm	12 Mo	NSAID 0.2% CHX
Von Arx et al, 2007	Prospective of case series	Switzerland	185 teeth	1. Sulcular incision (n=125) 2. Papilla-based incision (n=30) 3. Submarginal incision (n=30)	Amount of recession Clinical measurement using periodontal probe	At 1 year, buccal SI: 0.42 ± 0.69 mm PBI: 0.31 ± 0.49 mm SMI: 0.05 ± 0.61mm At 1 year, lingual SI: 0.31 ± 0.83 mm PBI: 0.06 ± 0.63 mm SMI: 0.14 ± 0.52 mm	12 Mo	NSAID 0.2% CHX Antibiotics Smoking
Kreisler et al, 2009	NRCT	Germany	n= 81 F (n=50) M (n=31) 44Y	1. Sulcular incision (n=65) 2. Submarginal incision (n=33)	Amount of recession Clinical measurement using periodontal probe	At 6 months, buccal SI: 0.3 ± 0.6 mm SMI: 0.5 ± 1.1	6 Mo	NSAID Glucocorticoid Antibiotics
Del Fabbro et al, 2009	RCT	Italy	n= 40 F (n=23) M (n=17) 22. 59Y	1. Sulcular Incision (n=19) 2. Papilla-based incision (n=19)	1. Pain 2. Swelling 3. Functional activities: chewing, talking, sleeping, phonetics, daily routine, missed work, bleeding, nausea, bad taste and breath Questionnaire Pain: ( VAS ) scale Others: 5-point Likert type scale	Pain and drug intake significantly less with PBI from day 3 VAS: SI: 75 PBI: 55 Swelling significantly less with PBI Day 1: severe swelling SI:15.8% PBI: 0% Day 2: severe swelling SI: 42. 1% PBI: 5.3% Chewing impairment significantly higher with SI Day 1: severe impairment SI: 42.1% PBI: 26.3% Day 2: severe impairment SI: 15.8 % PBI: 0% Others: Similar	Daily for 7 days	0.2% CHX Ice pack NSAID Smoking
Ahmed et al, 2013	RCT	India	n=20 F (n=11) M (n=9) 12–40Y	1. Submarginal incision. (n=10) 2. Sulcular incision (n=10)	1. Pain 2. Amount of recession Pain: (VAS) scale Recession: index of recession by Smith	VAS SMI: 55.3 ±3.31 SI: 58.4 ±4.8 Recession more with SI	Pain: hourly for 12 h Others: 24 h, 3, 7, 15 days,1 Mo	Antibiotics NSAID
Taschier et al, 2014	NRCT	Italy	n= 24 >18Y	1. Papilla-based incision(n=10) 2. Sulcular incision (n=10)	1. Amount of recession determined by comparing to pre-operative resin model	At 2 weeks SI: 2. 05(M), 1. 80(D) mm PBI: 0.10(M), 0.20 (D) mm At 6 months SI: 0.40(M), 0.45(D) mm PBI: 0.20 (M), 0.10 (D) mm	2 weeks 6 Mo	0.2% CHX NSAID Smoking
Taschier et al, 2016	NRCT	Italy	n= 24 >18Y	1. Papilla-based incision (n=10) 2. Sulcular incision (n=11)	1. Amount of recession determined by comparing to pre-operative resin model	SI: 0.05 ± 0.15 (M), 0.05± 0.15 (D) PBI: 0.00 ± 0.00 (M), 0.10± 0.32 (D)	12 Mo	0.2% CHX NSAID Smoking
Dimova et al, 2016	NRCT	Macedonia	n= 60 F (n=31) M (n=29) 35-43Y	1. Gingival flap design (triangular or envelope) 2. Semilunar flap design	1. Pain 2. Swelling 3. Functional activities: mouth opening, chewing, talking, sleeping, daily routine (activity), bleeding, nausea, bad taste and breath Questionnaire 5-point Likert type scale	Pain and drug intake statistically significantly higher with GFD on day 3 GFD: 4.1 ± 0.9 SFD: 3.7 ± 1. 3 Sig. difficulty in mouth opening, with SFD on day 1 GFD: 3.9 ± 1. 9 SFD: 2.1 ± 1.2 Sig. difficulty in mastication with SFD on day 1 GFD: 2.9 ± 1.9 SFD: 2.2 ± 1.9 Sig. difficulty in ability to speak more with SFD on day 1 GFD: 2. 5± 0.8 SFD: 1. 7 ± 0.3	Daily for 7 days	NSAID

CHX: chlorhexidine; D: distal; F: female; GFD: gingival flap design; h: hours; M: male; Me: mesial; MI: marginal incision; mm: millimeter; Mo: month; NRCT: Non-randomised controlled clinical trial; NSAID: Nonsteroidal anti-inflammatory drug; RCT: randomised controlled clinical trial; PBI: Papilla-based incision; SFD: semilunar flap design; Sig: significant; SI: sulcular incision; SMI: submarginal incision; VAS: visual analog scale; Y: years.

The extracted data demonstrates the amount of recession after surgical endodontic treatment using either a cast model or clinical examination. Data also revealed OHRQoL measurement using the VAS or the 5-point Likert scale.

### Risk of Bias Quality Assessment

Two randomised clinical trials^2,11^ were assessed using the Cochrane Collaboration’s Risk of Bias tool. The random sequence generation was adequately performed in one study.^11^ The assessor was not adequately blinded in either trial. Overall, both randomised clinical trials were judged to have a high risk of bias and could not be included in the meta-analysis (Table 3).

**Table 3:**
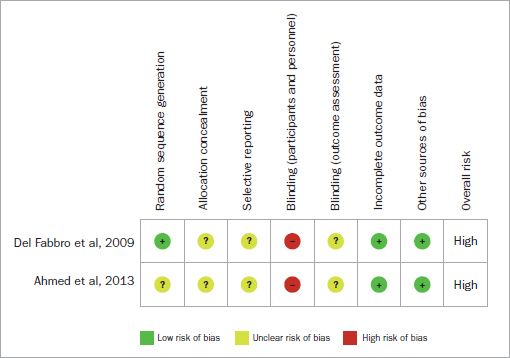
Risk of bias summary: review authors’ judgments about each risk of bias item for each included randomised controlled clinical trial

The 8 included nonrandomised clinical trials studies were qualitatively analysed using the Newcastle-Ottawa Quality Assessment scale. According to the methodological quality assessment, one study was judged to be poor quality,^19^ two studies were fair quality^13,54^ whereas the other five studies were considered good quality (Table 4).^27,46,47,53,56^

**Table 4:** Risk of bias summary: review authors’ judgments about each risk of bias item for each included non-randomised controlled clinical trial

	Selection	Comparability	Outcome	Overall score, Newcastle-Ottawa scale
Grung, 1973	*		*	2 stars
Velvart et al, 2003	* * *		* *	5 stars
Velvart et al, 2004	* * *		* * *	6 stars
Von Arx et al, 2007	* * *		* * *	6 stars
Kreisler et al, 2009	* * *		* * *	6 stars
Taschier et al, 2014	* * *	* *	* * *	8 stars
Taschier et al, 2016	* * *	* *	* * *	8 stars
Dimova et al, 2016	* *		*	3 stars

In the selection category of the Newcastle-Ottawa Quality Assessment scale, the author should mention if the participants represented the community. This analysis found that the participants did not represent the whole community in any of the enrolled articles due to the surgical intervention. All articles except one^19^ reported that both groups were drawn from the same community. All articles used surgical records. All articles except two^13,19^ stated that the outcomes of interest – gingival recession or OHRQoL – were not mentioned in the study.

For the comparability parameter, only two studies^46,47^ were comparable. These two studies reported the control for age, sex, and marital status, as well as other confounding factors, such as smoking.

For the outcome category, all studies except one^13^ assessed outcomes through clinical examination. Follow-up reports were completed for all papers, except for two articles^13,19^ reporting over a period less than six months (cut-off). All articles reported complete follow-up of all subjects enrolled in the study, or ≤ 20% of ‘lost to follow-up’, except two articles^13,19^ which did not mention that.

### Primary Outcome: The Impact of Incision Type on Patient Satisfaction Including Gingival Recession

Gingival recession was evaluated in 8 articles.^2,19,27,46, 47,53,54,56^ Clinically evaluation was performed using a periodontal probe^2,3,27^ or using study models.^19,54^ Velvart et al^54^ reported that gingival recession was statistically significantly greater in sulcular incisions compared to papilla-based incisions after post 1 month, 3 months,^54^ and 12 months^53^ (SI: 0.98 ± 0.75 mm; PBI: 0.06 ± 0.21 mm at 12 months) using a plaster cast. Taschieri et al^47^ reported that gingival recession was statistically significantly greater in sulcular incisions compared to papilla-based incisions over a period of 2 weeks.^47^ However, there was no statistically significant difference between the two groups over 6 months^47^ and 12 months,^46^ (SI: 0.10 ± 0.32 mm; PBI: 0.05 ± 0.15 mm at 12 months) as shown by reference to a custom-made resin model prepared before the surgery. Two more studies reported that gingival recession is statistically significantly greater in sulcular incisions compared to submarginal incisions at 6 months (SI: 0.5 ± 1.1 mm, submarginal: 0.3 ± 0.6 mm)^27^ and 12 months (SI: 0.42 ± 0.69 mm, submarginal: 0.05 ± 0.61 mm)^56^ according to clinical examination and using a periodontal probe. Ahmed et al^2^ and Grung^19^ reported more recession with sulcular incisions using either Smith’s recession index or a plaster model, respectively.

### Secondary Outcome: The Impact of Incision Design on Quality of Life

OHRQoL was assessed in three of the included studies.^2,11,13^ Two studies found more pain associated with the sulcular incision design compared to other incision types.^2,11^ Del Fabbro et al^11^ assessed the level of pain and drug intake, swelling, and chewing ability using the VAS and 5-point likert scale. They concluded that the quality of life was significantly higher in the papilla-based incision group compared to that of the sulcular incision group. The pain level and drug intake were statistically significantly less on the third day in the papilla-based incision group (VAS: 55) compared to the sulcular incision group (VAS: 75). Severe swelling was reported on the first two days in only 5.3% with papilla-based incisions, compared to 57.9% with sulcular incisions. Severe chewing impairment was reported on the first two days in only 26.3% with papilla-based incisions vs 57.9% with sulcular incisions. Using the VAS, Ahmed et al^2^ reported that pain was statistically insignificantly higher with sulcular incisions (58.4 ± 4.8) than with submarginal incisions (55.3 ± 3.31). Dimova et al^13^ compared two flap designs and reported that the semilunar flap caused less postoperative pain, but more difficulty in mouth opening, mastication, and ability to speak, compared to the gingival flap design.

### Quantitative Analysis (Meta-analysis)

**Fig 2:**
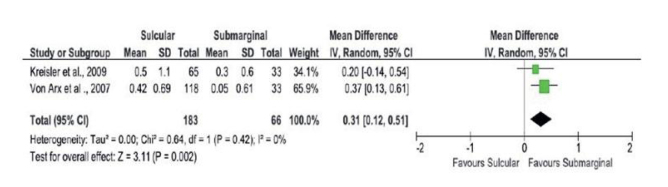
Forest plot comparing sulcular vs submarginal incisions. No heterogeneity among study outcomes was found.

The two eligible studies^27,56^ deemed to be of high methodological quality and of low risk of bias were included in the meta-analysis. A forest plot was constructed in relation to the gingival recession. Two studies^27,56^ showed considerable gingival recession associated with the sulcular incision design (p = 0.002). Figure 2 reveals the forest plot of differences in gingival recession between sulcular and submarginal incisions. Subjects with submarginal incisions showed a favourable outcome; the mean difference in the gingival recession was 0.31 mm (0.12 – 0.51), and no heterogeneity was found between these studies (Q = 0.64, df = 1, p = 0.42, I^2^ = 0%).

## Discussion

Evidence-based dentistry encourages systematic analysis of scientific evidence by clarifying or reviewing controversial dental issues.^42^ The current study is a systematic review of evidence, assessing the impact of incision designs on gingival recession, patient satisfaction, and quality of life following endodontic surgery.

Ten articles reporting clinical trials that fulfilled the inclusion criteria were identified. Only two studies assessed the impact of flap designs on the quality of life and found more pain associated with the sulcular incision design. The results based on meta-analysis found statstically significant gingival recession associated with the sulcular incision design: it was observed to increase the risk of gingival recession and decrease the patients’ quality of life more than other types of incisions.

The factors responsible for postoperative gingival recession are not fully understood. There are patient-related and clinician-related factors. Patient’s pre- and post- surgical oral hygiene, quality of periodontal tissues such as the shape of the gingival papilla, size of the lesion, and healing potential might influence surgery outcomes. Moreover, a full-thickness flap allows complete mobilisation of interdental papilla, causing papillary damage and necrosis of tissues due to insufficient blood supply.^16,45,58^ Insufficient adaptation of the papilla to the underlying bone at the time of flap re-approximation is considered to cause gingival recession. Additionally, the force levels applied during flap reflection are reported to have a negative influence on the gingival margin.^22,30^ Velvart^52^ reported that scalpel size, needle size, type of suture material, number of sutures placed and day of suture removal may also increase the risk of gingival recession. Hence, the majority of the factors responsible for post-surgical gingival recession may be unrelated to the flap design.

All the flap incisions showed that factors such as age, gender, smoking, site of operation and size of the lesion had no influence on the gingival recession. One study reported no significant difference between non-smokers and smokers.^55^ In contrast, few studies reported conflicting results regarding the effect of smoking on postoperative pain and swelling.^2,17^ This variation could be due to the differences in periodontal and endodontic surgeries.^31,41,48^ Periodontal surgery involves healing of inflamed epithelial tissue by secondary intention, in contrast to apical surgery. A review by Duncan et al^14^ reported no specific relationship between smoking and surgical endodontics.

OHRQoL is associated with functional factors, psychological factors, social factors, and the experience of pain.^4,24,25,43^ This study focuses on postsurgical complications such as pain, swelling, and impaired chewing, as well as esthetic outcomes. Poor preoperative oral hygiene may negatively impact the severity of pain and swelling after periapical surgery.^17^ However, one study found no statistically significant influence of the above-mentioned factors.^38^ Modern endodontic surgery involves the use of magnifying lenses during the handling of soft tissues, facilitating successful treatment and OHRQoL.^51^

The criterion of conducting a 7-day follow-up for OHRQoL and one of at least 12 months for gingival recession was considered. All measures showed statistically significant changes in the OHRQoL during the first five postoperative days. The maximum pain intensity was recorded on the day of the operation. It started 3 to 5 hours after surgery and continued the whole day.^6,25,29^ In contrast, some authors have found maximum pain intensity on the day following surgery for three consecutive days,^8,49,50^ and swelling reaching a maximum 48 h after surgery.^6,29^ Goldman et al^18^ described the creeping of the gingival tissue, which occurs between a month and a year after periodontal surgery, with no significant changes after a year.^21,32^

This systematic review had some limitations. First, the nonrandomised design in eight studies had a significant bias, although both groups were statistically compared at baseline. Limiting the variability between the groups with the clinical trials increases the risk of bias. Second, the sample size in all considered studies was relatively small, although sample size calculations were done. Operator experience should be a significant factor, especially when evaluating the external validity. Finally, outcomes pertaining to gingival recession measurement and OHRQoL, as well as duration of follow-up, were variable. More well-constructed studies with low risk of bias and a larger sample size are needed in the future to provide definitive clinical guidance.

## Conclusions

This review is the first to discuss the effect of flap incision on gingival recession and OHRQoL following endodontic surgery. It was concluded that sulcular incisions may have an unfavourable impact, with gingival recession statistically significantly associated with sulcular incision and reduced oral health related quality of life.
